# Supplementary material for: The Extracellular Matrix Vitalizer RATM Increased Skin Elasticity by Modulating Mitochondrial Function in Aged Animal Skin
Source: Antioxidants (Basel). 2023 Mar 11;12(3):694. doi: 10.3390/antiox12030694 (PMC10044720; doi:10.3390/antiox12030694)
Supplement: Supplementary file 1 [file antioxidants-12-00694-s001.zip › antioxidants-2249537-supplementary.pdf]

antioxidants

MDPI

*Article*

# The Extracellular Matrix Vitalizer RA™ Increased Skin Elasticity by Modulating Mitochondrial Function in Aged Animal Skin

**Kyung-A Byun [1], Seyeon Oh [2], Sosorburam Batsukh [1,2], Min Jeong Kim [3], Je Hyuk Lee [4], Hyun Jun Park [5], Moon Suk Chung [6], Kuk Hui Son [7,*], and Kyunghee Byun [1,2,*]**

[1] Department of Anatomy & Cell Biology, Gachon University College of Medicine, Incheon 21936, Republic of Korea
[2] Functional Cellular Networks Laboratory, Department of Medicine, Graduate School and Lee Gil Ya Cancer and Diabetes Institute, College of Medicine, Gachon University, Incheon 21999, Republic of Korea
[3] Mihana Clinic, Gyeonggi 17051, Republic of Korea
[4] Doctorbom Clinic, Seoul 06614, Republic of Korea
[5] Maylin Anti-Aging Clinic, Seoul 06005, Republic of Korea
[6] I`ll Global Co., Inc., Seoul 06532, Republic of Korea
[7] Department of Thoracic and Cardiovascular Surgery, Gachon University Gil Medical Center, Gachon University, Incheon 21565, Republic of Korea
* Correspondence: dr632@gilhospital.com (K.H.S.); khbyun1@gachon.ac.kr (K.B.); Tel.: +82-32-460-3666 (K.H.S.); +82-32-899-6511 (K.B.)

**Table S1. List of reagent required for RA**

| Reagent | Company | Catalog no. | Dilution rate |
|---|---|---|---|
| Ascorbic Acid | Sigma-Aldrich | A92902 | 0.25% |
| Niacinamide | Sigma-Aldrich | 72340 | 0.25% |
| Coenzyme A | Sigma-Aldrich | C4282 | 0.00002% |
| Glutathione | Sigma-Aldrich | G6013 | 0.00005% |
| Sodium Hyaluronate | Daejung Chemicals & Metals Co. Ltd. | 7848-4440 | 0.20% |

**Table S2. List of antibodies for western blotting (WB), immunohistochemistry (IHC), and enzyme-linked immunosorbent assay (ELISA).**

| Index | Antibody (host) | Company | Catalog no. | Dilution rate |
|---|---|---|---|---|
| ELISA | 8-OHdG (Mouse) | Gene Tex | GTX41980 | 1:500 |
| | COL1A1 (Mouse) | Santa cruz biotechnology | sc-293182 | 1:500 |
| | COL3A1 (Rabbit) | Bioss Antibodies Inc. | BS-0549R | 1:500 |
| WB | SOD2 (Rabbit) | Abcam | ab68155 | 1:1,000 |
| | PGC-1$\alpha$ (Rabbit) | Novus biologicals | NB100-1750 | 1:1,000 |
| | COX1 (Mouse) | Invitrogen | 358100 | 1:4,000 |
| | SDHA (Rabbit) | Affinity | DF7043 | 1:4,000 |
| | DRP1 (Mouse) | Abclonal | A2586 | 1:4,000 |
| | FIS1 (Rabbit) | Mybiosource | MBS9603233 | 1:1,000 |
| | OPA1 (Rabbit) | Affinity | DF8587 | 1:500 |
| | MFN2 (Mouse) | Abclonal | A12771 | 1:4,000 |
| | TGF-$\beta$ (Rabbit) | abcam | ab64715 | 1:4,000 |
| | CTGF (Rabbit) | Santa cruz biotechnology | sc-373936 | 1:1,000 |
| | $\alpha$-SMA (Rabbit) | invitrogen | 14-9760-82 | 1:4,000 |
| | $\beta$-actin (Rabbit) | Cell signaling technology | 4967s | 1:1,000 |
| IHC | Laminin (Rabbit) | Novus biologicals | NB300-144 | 1:1,000 |
| | Nidogen (Mouse) | Santa cruz biotechnology | sc-47773 | 1:50 |

**Table S3. List of primers used for quantitative real-time polymerase chain reaction.**

| Gene (Host) | | Primer |
|---|---|---|
| *ACTB (Human)* | Forward | 5'-GGGACCTGACTGACTACCTCAT-3′ |
| | Reverse | 5'-CCTTAATGTCACGCACGATTT-3′ |
| *PGC-1α (Human)* | Forward | 5'-ACTCAGCAAGTCCTCAGTCCTC-3′ |
| | Reverse | 5'-TTGACTGGCAATAGTCATGGTC-3′ |
| *COX1 (Human)* | Forward | 5'-GCTCGTAGGAGAGAAGGAGATG-3′ |
| | Reverse | 5'-CAGTCCAGGGTAGAACTCCAAC-3′ |
| *SDHA (Human)* | Forward | 5'-GAAAGGTTTATGGAGCGATACG-3′ |
| | Reverse | 5'-CTTCTCGGATCTCCAGAGTCAT-3′ |
| *DRP1 (Human)* | Forward | 5'-AGGATCATTCAGCACTGTAGCA-3′ |
| | Reverse | 5'-TCACCACTTCAACTATGGCATC-3′ |
| *FIS1 (Human)* | Forward | 5'-GGACCTGCTGAAGTTTGAAAAG-3′ |
| | Reverse | 5'-GTACTCAAACTGCGTGCTCTTG-3′ |
| *OPA1 (Human)* | Forward | 5'-CGGGAGTTTGATCTTACCAAAG-3′ |
| | Reverse | 5'-ACGGTACAGCCTTCTTTCACAT-3′ |
| *MFN2 (Human)* | Forward | 5'-GTGTCTGTGCGGAGTCAGATAG-3′ |
| | Reverse | 5'-AGCACACAGCTTGTCACAGTTT-3′ |
| *P21 (Human)* | Forward | 5'-GGAGACTCTCAGGGTCGAAAA-3′ |
| | Reverse | 5'-GCTTCCTCTTGGAGAAGATCAG-3′ |
| *P16 (Human)* | Forward | 5'-CAACGCACCGAATAGTTACG-3′ |
| | Reverse | 5'-ATCTATGCGGGCATGGTTACT-3′ |
| *ACTB (Mouse)* | Forward | 5'-CCGTAAAGACCTCTATGCCAAC-3′ |
| | Reverse | 5'-GCAGTAATCTCCTTCTGCATCC-3′ |
| *P21 (Mouse)* | Forward | 5'-GACAAGAGGCCCAGTACTTCC-3′ |
| | Reverse | 5'-CTCAGACACCAGAGTGCAAGAC-3′ |
| *P16 (Mouse)* | Forward | 5'-GTACCCCGATTCAGGTGATG-3′ |
| | Reverse | 5'-AGTTCGAATCTGCACCGTAGTT-3′ |
| *NF-κB (Mouse)* | Forward | 5'-GGGACCTGACTGACTACCTCAT-3′ |
| | Reverse | 5'-CCTTAATGTCACGCACGATTT-3′ |
| *AP-1 (Mouse)* | Forward | 5'-CTCGGCTAGAGGAAAAAGTGAA-3′ |
| | Reverse | 5'-CTTAAGCTGTGCCACCTGTTC-3′ |
| *MMP1 (Mouse)* | Forward | 5'- AATTCAGATCCTGAAACCCTGA -3′ |
| | Reverse | 5'- CCAACGAGGATTGTTGTGAGTA-3′ |
| *MMP2 (Mouse)* | Forward | 5'- AACAAGTATGAGAGCTGCACCA -3′ |
| | Reverse | 5'- ACTTCCGGTCATCATCGTAGTT -3′ |

| | | |
|---|---|---|
| *MMP3 (Mouse)* | Forward | 5'- GTCTTTGAAGCATTTGGGTTTC -3′ |
| | Reverse | 5'- TGCTCTTCAATATGTGGGTCAC -3′ |
| *MMP9 (Mouse)* | Forward | 5'- TCACACGACATCTTCCAGTACC -3′ |
| | Reverse | 5'- CACCTCATTTTGGAAACTCACA -3′ |

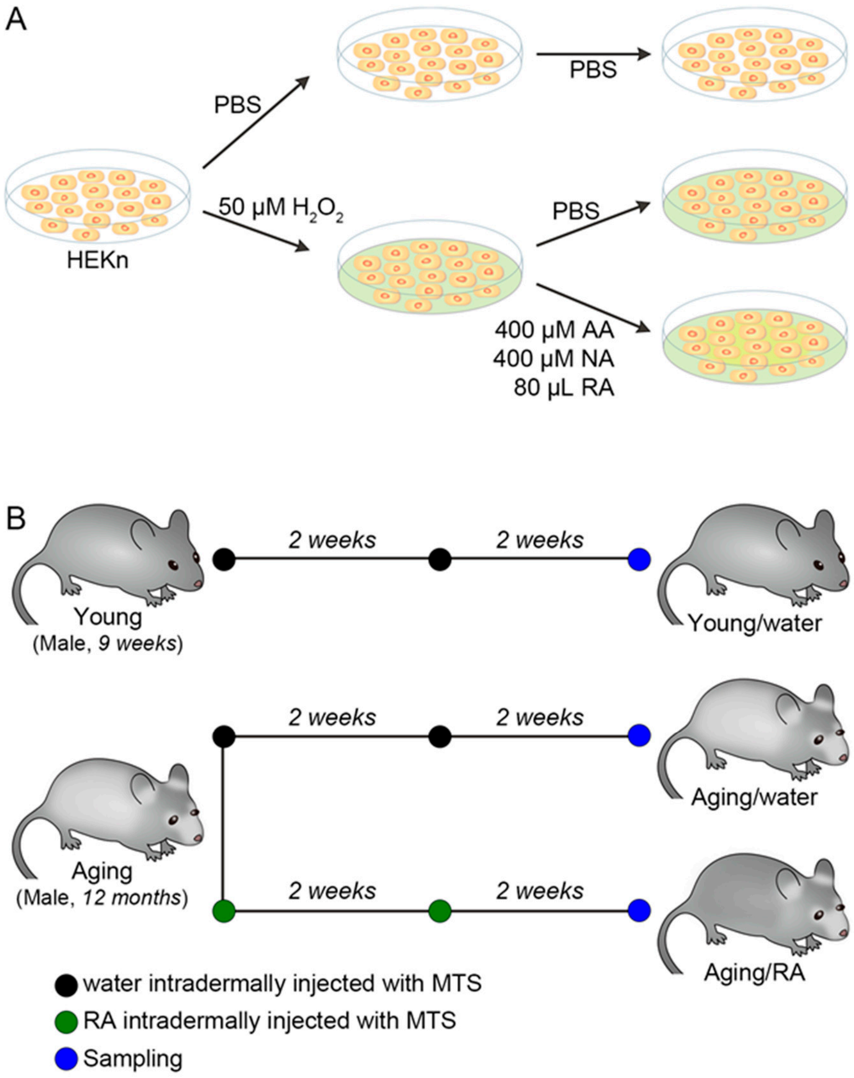


**Figure S1. Schematic diagram of the *in vitro* (A) and *in vivo* (B) experiment in this study.** AA; ascorbic acid, HEKn; human primary epidermal keratinocytes, MTS; microneedle therapy system, NA; niacinamide, PBS; phosphate-buffered saline.
